# Light-Emitting Diode Array with Optical Linear Detector Enables High-Throughput Differential Single-Cell Dielectrophoretic Analysis

**DOI:** 10.3390/s24248071

**Published:** 2024-12-18

**Authors:** Emerich Kovacs, Behnam Arzang, Elham Salimi, Michael Butler, Greg E. Bridges, Douglas J. Thomson

**Affiliations:** 1 Department of Electrical and Computer Engineering, University of Manitoba, Winnipeg, MB R3T 5V6, Canada; kovacse3@myumanitoba.ca (E.K.); arzhang1@myumanitoba.ca (B.A.); elham.salimi@umanitoba.ca (E.S.); gregory.bridges@umanitoba.ca (G.E.B.); 2 Department of Microbiology, University of Manitoba, Winnipeg, MB R3T 5V6, Canada; michael.butler@nibrt.ie; 3 National Institute for Bioprocessing Research and Training, A94 X099 Dublin, Ireland

**Keywords:** dielectrophoresis, single-cell sensor, differential detection, lens-free, dielectric spectrum, in-flow analysis

## Abstract

This paper presents a lens-free imaging approach utilizing an array of light sources, capable of measuring the dielectric properties of many particles simultaneously. This method employs coplanar electrodes to induce velocity changes in flowing particles through dielectrophoretic forces, allowing the inference of individual particle properties from differential velocity changes. Both positive and negative forces are detectable. The light source utilized in this system is composed of LEDs with a wavelength of 470 nm, while detection is performed using a 256-element optical array detector. Measurements with 10 μm polystyrene beads demonstrate this method can resolve changes equivalent to a Clausius–Mossotti factor of 0.18. Simulations in this work, using values from the literature, predict that Clausius–Mossotti factor differences of 0.18 are sufficient to differentiate viable from nonviable cells and cancerous from multidrug-resistant cancerous cells. We demonstrate that for Chinese hamster ovary (CHO) cells, the method can collect a dielectric response spectrum for a large number of cells in several minutes. We demonstrate that for CHO cells, Clausius–Mossotti factor differences of 0.18 can be discriminated. Due to its simple detection apparatus and the utilization of high-throughput, wide, clog-resistant channels, this method holds promise for a wide range of applications.

## 1. Introduction

Dielectrophoresis (DEP) is a promising technique for single-cell analysis, as numerous physiological changes in cells are known to cause significant dielectric changes. DEP has been applied for various purposes, such as identifying different cell types in blood analysis [1,2], manipulating single cells [3], identifying and isolating circulating tumor cells [4,5], differentiating cancerous cells from healthy ones [6,7,8,9,10], identifying different types of cultured tumor cells [3,11], characterizing breast cancer cells [12], separating and differentiating stem cells [13,14,15,16], tracking apoptosis [17,18,19,20,21,22,23], and monitoring the effects of nutrient deprivation [19,24,25].

When appropriate analysis conditions are applied, DEP analysis can measure changes in dielectric properties such as cytoplasm conductivity (ion concentration and mobility) or the cell membrane structure. For instance, apoptosis can substantially alter the ion concentrations in the cytoplasm and the effective surface area of the cell membrane, resulting in changes to cytoplasm conductivity and membrane capacitance [20,21,22,26]. The label-free nature of DEP gives it an advantage over other techniques, such as flow cytometry and magnetic bead attachment technologies, which require cells to be incubated with dyes or magnetic beads for cell characterization and separation. Other label-free dielectric-based techniques, including impedance-based analysis and electrorotation, also exist for single-cell analysis. Impedance-based analysis methods use electrodes within the channel to measure the impedance perturbation caused by passing cells [27,28,29]. Although impedance-based analysis can have throughput exceeding 100 cells/s, it requires corrections for background media dielectric properties to extract quantitative estimates of cell dielectric properties and uses small channels that are prone to clogging to enhance signals. Electrorotation is also a single-cell approach; however, its throughput is limited to approximately one cell per minute [30,31,32]. This work presents a label-free high-throughput DEP single-cell analysis method enabled by an LED optical source array with a linear array optical detector.

Cells are membrane-bound bodies filled with electrolytes and smaller organelles. They expend considerable energy to maintain specific ionic concentrations in the cytoplasm and to preserve the structure of the cell membrane. However, during disease states or periods of environmental stress, such as during starvation, the ionic equilibrium and the membrane structure can become altered. These changes are reflected in corresponding changes to the cell’s compartmental dielectric properties and thus the cell’s overall polarizability. Therefore, dielectric analysis methods can also provide a platform to track cellular changes due to disease or environmental stresses [25,33]. Cell populations are heterogeneous due to differences in cell cycle phase or non-uniform dynamic response to stress factors. It is important to distinguish these subpopulations. Single-cell methods are needed to monitor the emergence and growth of these subpopulations [34].

The usefulness of any dielectric analysis method will in part be determined by how small a change in cell physiology can be detected. The relationship between cell physiology and dielectric response depends on many factors. For example, there can be changes in the cytoplasm or cell membrane due to disease states or environmental stress. The actual situation is complex and most simplified models use changes in the Clausius–Mossotti factor (CMF). The CMF quantifies the dipole moment induced in a suspended cell compared to the dipole moment that would be induced in a volume of suspension medium equal in volume to the cell [27,35]. The CMF change that can be detected is dependent on factors such as cell size and medium conductivity. However, in general, the smaller the change in the CMF that can be detected, the more sensitive an analysis method will be to changes in cell physiology. The most sensitive single-cell analysis methods use in-flow differential impedance or dielectrophoresis [23,27,28,36,37]. Differential impedance approaches, however, require small analysis sensing aperture to obtain usable signals and these types of devices are much more prone to clogging. In contrast, in-flow dielectrophoresis only requires a local field gradient and a method of measuring the cell’s translational response and this does not inherently require a small analysis sensing aperture.

In-flow single-cell dielectrophoresis analysis has been achieved using several detection methods, which can be broadly categorized as electronic or optical. Single-cell electronic detection of dielectrophoretic-induced translational motion has been accomplished using microwave interferometers and CMOS ring oscillators [23,38,39]. Among these, microwave interferometry is the most sensitive and can measure translational motions as small as 0.1 micrometers. However, single-cell electronic detection has limited throughput in a flow configuration, as typically only one cell can be detected at a time within the analysis volume.

Optical methods rely on measuring translational motion near field gradients created by micropatterned electrodes or electrode arrays on an insulating surface. In this manner, dielectrophoretic actuation is applied in parallel to a large number of cells and optical detection is used to measure individual cell response [20,40]. In dielectrophoretic field flow fractionation (DEP-FFF), the velocity of a particle is determined after the steady-state condition has been reached in a flow channel [14,41]. The dielectric properties of cells are often inferred using micropatterned electrodes on an insulating surface, in combination with a microscope and a camera to track the particle’s position [42,43]. In this approach, individual cells can be tracked using an optical microscope through multiple frames to give a measure of particle position versus time. Using the proximity to the electrode, the local field gradients can be estimated and used to infer the cell’s dielectric properties [39,44]. In addition, some methods extract the dielectric response of a population of cells by measuring the transmission of light through cell mixtures in the vicinity of electrodes [45,46]. These methods are rapid and can extract a 20-frequency spectral response in 10s of seconds [45,46]. However, these methods are for a population of cells and do not determine the cell-by-cell dielectric response.

An alternative to microscopy-based optical detection is the use of linear optical array detection of dielectrophoresis-induced motion of cells flowing over coplanar electrodes on an insulating surface [47]. That method improves throughput and resolution but measures cells only after actuation and thus suffers from errors at the single-cell level due to the unknown position and velocity of the cell before dielectrophoresis-induced motion.

In this work, a multiple-optical-source LED array combined with a simple lens-free linear optical array detector achieves high throughput with in-flow single-cell high-resolution differential dielectrophoresis. This approach is useful for discriminating between cell phenotypes without the need for dyes at throughputs of tens of cells per second. The relatively high throughput allows the dielectric response spectra for heterogenous cell populations to be measured in periods of minutes.

The next section calculates the sensitivity to dielectric changes (CMF) needed for several typical use cases and cell types. These calculations include the effects of cell-to-cell variability in the model parameters, in line with those observed in the literature. These calculations can be used to estimate what CMF resolution is required for typical cell analysis applications. The following sections describe the apparatus and sample preparation. This is followed by a results section with an analysis of both polystyrene beads and Chinese hamster ovary (CHO) cells. The discrimination resolution achieved here is compared with the model calculations. Finally, a discussion and conclusions are provided.

### Predictions of Dielectric Changes in Cells Due to Disease States or Environmental Stress

In this section, model calculations are used to predict the CMF resolution required for several typical applications. This analysis applies to any technique that uses DEP forces and not just to the technique presented in this work. As discussed previously, the Clausius–Mossotti factor (CMF), is used in simplified dielectric response models of the cell within a surrounding medium. To predict the required CMF measurement resolution needed for several typical cell analysis applications, we use model calculations of CMF distributions versus DEP frequency, including cell-to-cell variability in the model parameters. In this section, we use model calculations based on the measured cell dielectric properties of unaltered and altered cells to calculate the expected CMF distributions versus frequency. Using these CMF distributions versus frequency spectra, we can identify the CMF resolution and also optimal frequencies required for different applications, such as identifying drug-resistant cancer cells and nonviable cells. We selected cases from the literature where the radius, cytoplasm conductivity, and membrane capacitance were measured for both altered and unaltered cells. With this information and a suitable medium conductivity, we calculated the CMF distribution spectra.

To predict the cell’s dielectric response in suspension, it is most common to model the cell as a sphere composed of concentric spherical shells of dielectric material [48,49]. Two models are typically used: the single-shell model and double-shell model. In the single-shell model, large organelles within the cytosol, such as the nucleus or membrane-bound organelles, are incorporated, resulting in effective cytoplasm conductivity ($\sigma_{cyt}$) and permittivity ($\varepsilon_{cyt}$). The double-shell structure expands the model to include a membrane-bound region within the cytoplasm so that cells with a nucleus, particularly cells such as lymphocytes with a relatively large nucleus, can be modeled more precisely. However, the double-shell model parameters have not been measured for a large number of cell lines. The double-shell model incorporates elements from the nucleoplasm ($\varepsilon_{n}$, $\sigma_{n}$), nuclear envelope ($\varepsilon_{ne}$, $\sigma_{ne}$), cytoplasm ($\varepsilon_{c}$, $\sigma_{c}$), and plasma membrane ($\varepsilon_{m}$, $\sigma_{m}$). Equation set 1 shows how to calculate the effective complex permittivity of the entire multi-shell cell.

$${\hat{\varepsilon }}_{cell}={\hat{\varepsilon }}_{m}\frac{2\left(1-v_{1}\right)+\left(1+2v_{1}\right)E_{1}}{2+v_{1}+(1-v_{1}{)E}_{1}},$$

(1) $$E_{1}=\frac{{\hat{\varepsilon }}_{c}}{{\hat{\varepsilon }}_{m}}\frac{2\left(1-v_{2}\right)+\left(1+2v_{2}\right)E_{2}}{2+v_{2}+\left(1-v_{2}\right)E_{2}},$$

$$E_{2}=\frac{{\hat{\varepsilon }}_{n}}{{\hat{\varepsilon }}_{ne}}\frac{2\left(1-v_{3}\right)+\left(1+2v_{3}\right)E_{3}}{2+v_{3}+(1-v_{3})E_{3}},$$

where $E_{3}={\hat{\varepsilon }}_{n}/{\hat{\varepsilon }}_{ne}$ and ε^i=εi−jσiω, in which ω is angular frequency. In the above, $\nu_{1}={\left(1-d/R\right)}^{3}$, $\nu_{2}={\left(R_{n}/(R-d)\right)}^{3}$ and $\nu_{3}={\left(1-d_{n}/R_{n}\right)}^{3}$. Here, R_n_ is the radius of the nucleus, d_n_ is the thickness of the nuclear envelope, d is the thickness of the plasma membrane, and R is the radius of the cell [49].

The difference in complex permittivity between the cell and the medium results in a net-induced dipole due to the presence of the cells. The CMF quantifies the dipole moment induced in a suspended cell, compared to the dipole moment that would be induced in a volume of suspension medium equal in volume to the cell. For a spherical cell, the CMF can be expressed as follows:

(2) $$K_{CM}\left(\omega \right)=\frac{{\hat{\varepsilon }}_{cell}-{\hat{\varepsilon }}_{m}}{{\hat{\varepsilon }}_{cell}+2{\hat{\varepsilon }}_{m}}$$

For spherical particles, the relationship between the CMF (K_CM_) and the dielectrophoretic forces can be calculated using Equation (3):

(3) $${\vec{F}}_{DEP}=\frac{3}{2}V_{c}\varepsilon_{m}Re\left\{K_{CM}\left(f_{DEP}\right)\right\}𝛻{\left|{\bar{E}}_{rms}^{DEP}\left(r\right)\right|}^{2},$$

where V_C_ is the volume of the particle, ε_m_ is the permittivity of the medium, E is the field generated by the DEP electrodes, and *f_DEP_* is the frequency of the DEP potential.

**Figure 1:**
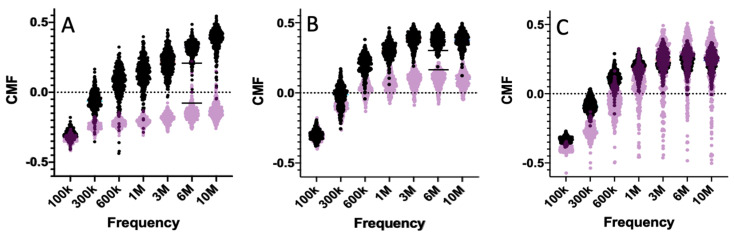
Violin plots from a Monte Carlo simulation of the real part of the Clausius–Mossotti factor (CMF) versus frequency for both normal and altered cells. (**A**) Viable (black) and nonviable CHO cells, (**B**) K562 and K562R (black) cells, and (**C**) breast cancer cells (black) and monocytes (WBC). Horizontal lines are for the 10th and 90th percentiles for CHO and K562 cells at 6 MHz.

In Figure 1A, violin plots of the real part of the CMF spectra from 100 kHz to 10 MHz for viable and nonviable CHO cells are shown. In this type of plot, each observation or calculation is represented as a point, and no assumption is made about the distribution of the underlying population. The CMF has been calculated using a two-shell model [31]. In addition, a Monte Carlo simulation has been carried out assuming the cells within the same population have normal distributions with standard deviations of ±15% in cytoplasm conductivity, ±15% in membrane capacitance, and ±15% in cell radius. The standard deviations were chosen after cataloging several published works where single-cell parameters were measured [30,50,51,52,53,54,55,56]. These variations are intended to model the cell-to-cell variation in dielectric properties within a population of nominally identical cells and are not intended to account for experimental uncertainties. In these calculations, a medium conductivity of 0.2 S/m was used. Starting at 100 kHz, the spectra have close to the same value as all the cells have an intact insulating membrane; therefore, the dielectric response is similar to an insulating sphere for all cells. As frequency increases, the curves diverge due to the differences in cytoplasm conductivity, cell radius, and membrane capacitance. The frequency where the CMF crosses zero (dashed line) is called the cross-over frequency. Above the cross-over frequency, the CMF for cells with higher cytoplasm conductivity will increase above the CMF for cells with a lower cytoplasm conductivity. In the viable and nonviable CHO cells, this can be seen where the viable cell CMF rises above zero at frequencies of several hundred kilohertz, whereas the nonviable cell CMF remains below zero for the chosen media conductivity. To differentiate between viable and nonviable cells using a DEP measurement, the analysis technique must have resolution sufficient to distinguish between the two populations, including the cell-to-cell variation. In this model simulation, it was assumed that the 10th to 90th percentiles can be used to delineate each population. Using these percentiles, the 10th to 90th percentile CMF gap at 6 MHz is 0.25 (+0.20 to −0.05). The 10th and 90th percentiles are shown with a horizontal bar. Therefore, a dielectric analysis method would need a resolution of less than 0.25 CMF to be useful for single-cell viability determination. Another case where dielectric changes could be used to distinguish cell types is between drug-resistant and non-resistant cancer cells [57]. In glioblastoma phenotypes K652 and K652R (resistant), K652R has a higher cytoplasm conductivity, and hence, the CMF for K652R is expected to be greater than that of K652 for the chosen 0.2 S/m conductivity of the media [57]. The media conductivity of 0.2 S/m tends to yield a CMF of close to zero for the K652 cells but could be further optimized to increase the gap in CMF between the two populations. For a media conductivity of 0.2 S/m, using K652 and K652R at 6 MHz, the 10th to 90th percentile CMF gap is 0.14 (+0.30 to +0.16). The 10th and 90th percentiles are shown with a horizontal bar. Of course, not all physiological differences have CMF gaps that are detectable. For example, the dielectric difference between some breast cancer cells (SkBr3) and monocytes (type of white blood cell) yields CMF distributions with significant overlap, and single-cell dielectric differentiation is not possible using the 10th and 90th percentiles [29]. Analysis of the mean CMF has been carried out for other systems, and these are listed in Table 1. In the table, no allowance has been made for cell-to-cell variation. The span between the means for normal cells and altered cells ranges from 0.55 for viable compared to nonviable cells to far less than 0.1 for cases of ion pumps and ion pore inhibition.

## 2. Materials and Methods

**Figure 2:**
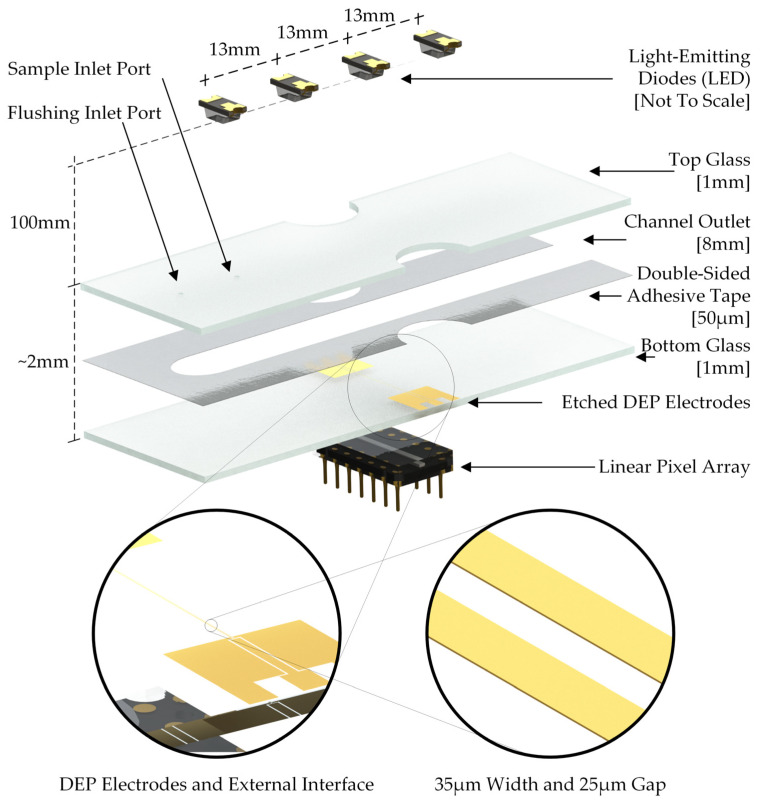
Schematic of the lensless DEP cytometer. Particles flow into the channel via the inlet port. The particles then flow down the channel to the channel outlet. At about halfway between the inlet and outlet, the particles pass over the electrodes that induce DEP motion. The LEDs above the channel illuminate the channel, including the detector array located below the electrodes. As a particle passes between an LED and the detector array, the light is partially blocked, resulting in a small decrease in the optical signal detected on the array.

The cytometer consists of three main components: the microfluidic channel, the illumination source, and the CMOS array sensor (Figure 2). The microfluidic channel used was 50 μm high, 13 mm wide, and 67 mm long. The channel was constructed by cutting the channel dimensions from double-sided tape (3M 9628FL). The thickness of the tape determined the height of the channel by pressing it between the two glass slides. A 1 or 3 mm hole was drilled in the top glass slide to act as the inlet port for the channel and the end of the channel was left open to act as the outlet. The bottom glass slide was patterned using photolithography to have two 35 μm wide gold coplanar electrodes with a 25 µm gap in between. The electrodes were used to create the non-uniform electric field necessary for DEP. The fluid moved through the channel by pressure-driven flow. The flow rate in the channel was controlled by the pressure generated by the height of the fluid reservoir at the inlet, which was placed just above the channel.

The microfluidic channel was illuminated by four blue LEDs (Wurth 150224BS73100) with a peak wavelength of 470 nm ± 15 nm. The LEDs were placed 100 mm above the channel and were spaced 13 mm apart. The 13 mm LED spacing was chosen to prevent overlap between the minima in the signal (see below) but was not optimized and could have been reduced to increase throughput. This LED was chosen due to its small emission area of approximately 150 µm. The small emission area allowed this LED to be used without a pinhole, which is common in other lens-free optical cytometers [61,62]. The diffracted light from the particles was captured by a linear optical sensor (AMS TSL1402R or Hamamatsu S14416), and the signal was digitized every 2 s. The sensor was oriented perpendicular to the flow so that the signal from many particles could be captured simultaneously. The sensor was located approximately 2 mm below the flow channel. This sensor consisted of 63.5 × 55.5 µm pixels with a 63.5 pitch, arranged in a linear array of 256 pixels. The voltage output signal from each pixel was digitized using an analog-to-digital converter (Analog Devices LTC2344-18).

**Figure 3:**
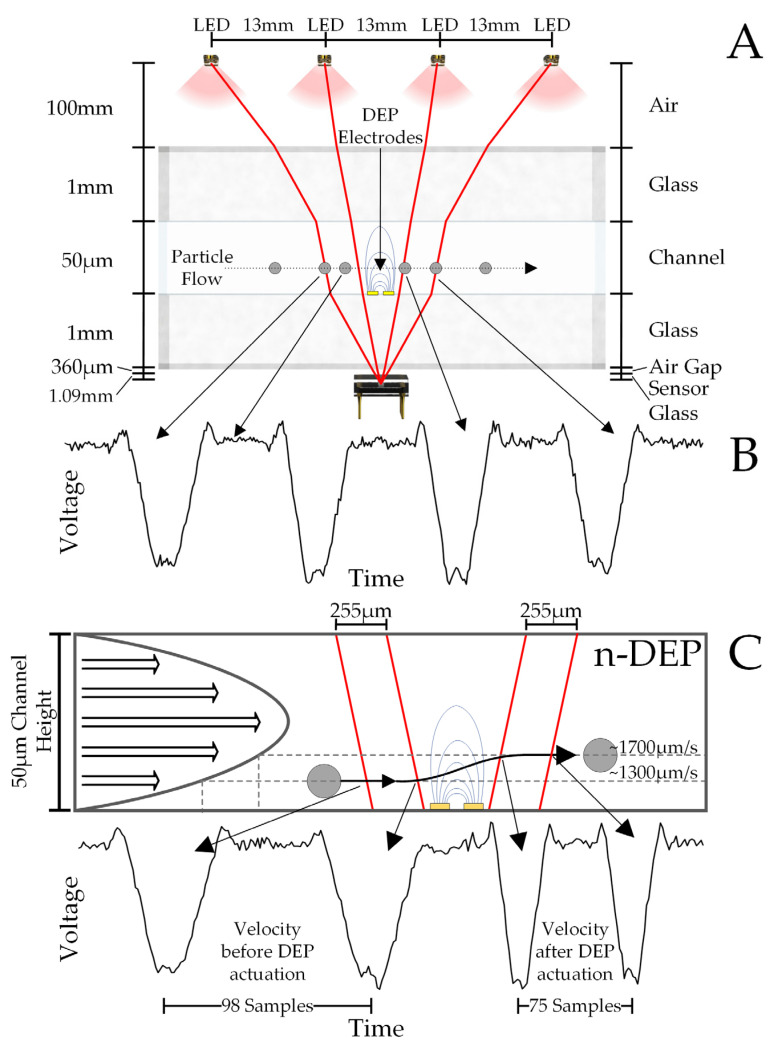
(**A**) Four LEDs illuminate the channel. When a particle passes between one LED and the detector, located below the channel, a minimum in the signal is produced. (**B**) Signal for a particle with a 4-LED light source experiencing no DEP force. (**C**) Typical signal for a particle passing with a repulsive n-DEP motion of the cell. The cell is repulsed to higher velocities after passing over the electrodes.

This DEP cytometer collects optical and DEP data simultaneously from particles flowing in the microfluidic channel. A mixture containing the particles flows through the microfluidic channel from the inlet to the outlet. The cytometer records the signal due to the averaged diffraction pattern generated as each particle passes through the light paths between each of the four LEDs and the optical detector (Figure 3A). Since each particle scatters the incident light, a minimum is generated for each of the four light paths as the particles flow past the electrodes. Therefore, each particle signal event consists of four minima. An example of a measured signal from a particle is shown in black in Figure 3B. The sequence of minima in the signal is used to determine the dielectric response of each particle.

The dielectric response can be inferred from the four minima due to the parabolic velocity profile of fluid flow in the channel, as shown on the left side of Figure 3C. Due to the parabolic velocity profile, the particle’s velocity is directly related to its height in the channel. The velocity decreases from the middle of the channel and towards the bottom of the channel. Initially, as each particle enters and flows in the channel, gravity will cause it to sink. Given sufficient time, the force of gravity will be balanced by lift forces and the particle will reach an equilibrium height and hence velocity [41,63,64,65,66].

The location of the LEDs, the electrodes, and the linear optical detector has been chosen such that the first two diffraction minima are generated before the DEP force is applied and the last two are generated after (Figure 3A,C). Depending on the dielectric properties of the particle, it will experience either a negative or positive DEP force (n-DEP, p-DEP). If a particle experiences n-DEP, it is pushed away from the electrodes and towards the middle of the channel which increases its velocity, as shown in Figure 3C. If a particle experiences p-DEP, it is pulled towards the electrodes and the bottom of the channel, which decreases its velocity [23,37]. Stokes’ drag forces limit the rate at which particles move due to DEP forces. Slower-moving particles experience DEP forces for a longer time. Therefore, for a given magnitude of DEP force, slower-moving particles will experience greater relative motion within the channel. An example of the diffraction pattern of a particle experiencing no DEP forces is shown in Figure 3B, and n-DEP is shown in Figure 3C. In the n-DEP case of Figure 3C, the third and fourth minima are recorded closer together compared to the first two minima, meaning the particle is now moving faster. This velocity information can be correlated with the Clausius–Mossotti factor. Due to the linear relationship of the CMF to the dielectrophoretic forces, the sign and magnitude of the velocity change indicate the sign and magnitude of the Clausius–Mossotti factor for the particle.

**Figure 4:**
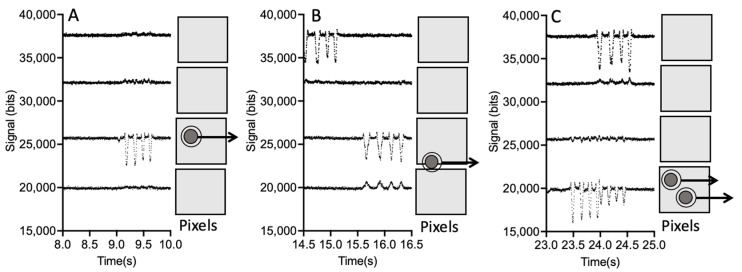
Signal traces for 4 adjacent pixels. (**A**) The cell is centered on one pixel and the signal comes from one pixel. (**B**) The cell overlaps two adjacent pixels and the signal occurs on both pixels. (**C**) The two cells are coincident in space, resulting in the signals overlapping in time.

The signals in Figure 3 are for a single pixel of the detector array. However, the detector array contains 256 pixels. An example of signals generated from 4 consecutive pixels as particles pass through the analysis region is shown in Figure 4. The LEDs are spaced 13 mm from center to center and are 100 mm above the detector. The flow channel is located approximately 2 mm above the optical sensor, with 1 mm being the sensor polymer cover and 1 mm being the bottom glass plate. Ray tracing was used to calculate the distance between dips as the particle flows through the channel. For the given geometry and materials used, the center-to-center spacing was calculated to be 255 μm. The vertical position of the particle in the channel is not important, since the channel depth is much less than the distance between the channel and the detector. For a particle traveling at a typical velocity of 1500 μm/s, the time between the particle crossing each light path and hence the time between the dips is 170 ms.

### Cell Preparation

The details of the cell growth and preparation are covered in detail elsewhere but are briefly outlined here [21]. The Chinese hamster ovary cells (CHODG44-EG2-hFc/clone 1A7), provided by Yves Durocher from Canada’s National Research Council, were grown in 250 mL shaker flasks and incubated at 37 °C with a 10% CO_2_ overlay on a shaker platform (120 rpm). The cells were passaged every 2–3 days with a seeding density of 2 × 10^5^ cells/mL in BioGro-CHO serum-free medium (BioGro Technologies, Winnipeg, MB, Canada) supplemented with 0.5 g/L yeast extract (BD, Sparks, MD, USA), 1 mM glutamine (Sigma, St. Louis, MO, USA), and 4 mM GlutaMax I (Invitrogen, Grand Island, NY, USA). Samples for DEP measurement were prepared by centrifuging and resuspending day 2 cells in a mix of BioGro CHO medium and low-conductivity medium [22.9 mM sucrose (Sigma), 16 mM glucose (Fisher), 1 mM CaCl_2_ (Fisher), and 16 mM Na_2_HPO_4_ (Fisher)] with a 1:15 ratio. The DEP medium has an osmotic pressure of 291 mOsm/kg and conductivity of 0.17 S/m. A 16 mL sample was obtained with a concentration of 2 × 10^5^ cells/mL.

## 3. Results

**Figure 5:**
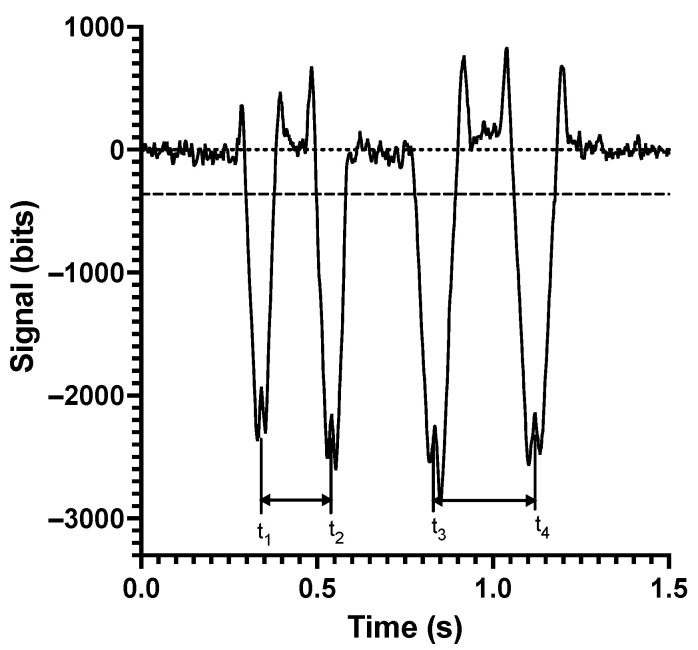
Analysis of a typical CHO cell signal. The cell velocity before and after is calculated using (t_2_ − t_1_) and (t_4_ − t_3_), respectively. In this case, (t_2_ − t_1_) is smaller than (t_4_ − t_3_), indicating p-DEP.

The measured signals versus time for 4 adjacent pixels of the 256-pixel array with 10 μm polystyrene beads (Polyscience) flowing in the channel are shown in Figure 4. Typically, the signals from 100 to 150 pixels are sampled. The groupings of four sequential dips due to the four sequential LED light paths are easily identified. An example of four sequential dips falling largely on one pixel is shown in Figure 4A. In Figure 4B, the particle is flowing closer to the adjacent pixel and the four sequential dips fall over two adjacent pixels. Figure 4C is an example of coincident particles, resulting in eight closely spaced signal dips. Coincident particles have been identified and excluded from the results below. In Figure 5, a close-up view of the four signal dips for a CHO cell is shown. For each dip, a threshold is used to find the rising and falling edge of the signal. The times of rising and falling edges are then averaged to give an estimate of the dip center. The center time of the four dips is used to calculate the velocity of the particle before and after the particle passes over the DEP electrodes. The CHO cell signal, in Figure 5, was taken under conditions where the CMF is positive. For a positive CMF, the particle is attracted to the electrodes and pulled towards a lower velocity flow, and hence, the velocity after the electrodes is lower. Greater time between dips after the electrodes compared to before the electrodes indicates the particle has reduced in velocity. Differential velocity is the difference in velocity before and after the electrodes.

**Figure 6:**
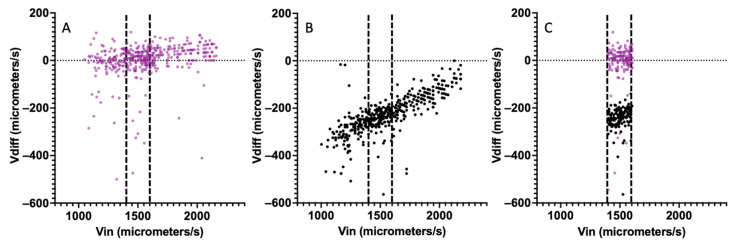
Scatter plots of incoming velocity against differential velocity for polystyrene microbeads. (**A**) With no DEP actuation, (**B**) with DEP actuation using a voltage of 2.5 Vpp at 1 MHz applied to the electrodes, and (**C**) filtered to include only incoming velocities between 1400 and 1600 μm/s for cases A and B.

Since the amount of time dielectrophoretic forces translate a particle depends on the particle velocity, a useful plot for analysis of the data is a plot of incoming velocity versus the differential velocity. Figure 6A is a plot of the incoming velocity versus the differential velocity for 10 μm polystyrene beads with no DEP actuation. The differential velocity is expected to be zero as the velocity before and after are expected to be the same. Figure 6A contains the extracted results from ~500 polystyrene beads and the mean differential velocity is close to 0 at −14 μm/s. Figure 6B shows the case for a field at 1 MHz (2.5 V peak to peak) where the DEP forces are repulsive [31]. The differential velocity is shifted negatively for the entire population. For polystyrene beads with slower incoming velocities, the particle experiences the repulsive forces for a longer time as it traverses the electrodes. Therefore, slower incoming particles have a larger change in velocity and hence the magnitude of differential velocity is greater. This is seen in Figure 6B where the shift is about ~300 μm/s at incoming velocities of 1200 μm/s. This change shrinks to ~100 μm/s at incoming velocities of 2000 μm/s. Particles can be grouped using the incoming velocity as a filter to identify particles that experience DEP forces for approximately the same period. Figure 6C is a scatter diagram where only particles with an incoming velocity between 1400 μm/s and 1600 μm/s are examined. This filtering still captures a large fraction of the particles but has the benefit of greatly reducing the variation in differential velocity due to incoming velocity.

**Figure 7:**
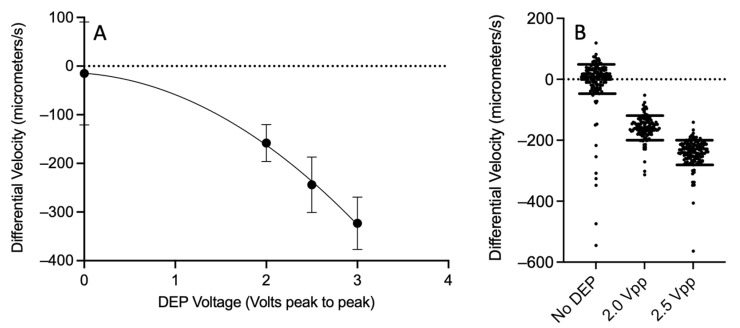
(**A**) Measured differential velocity for 10 μm diameter polystyrene beads in deionized water for different DEP voltages at 1 MHz. A parabolic best-fit line is also plotted. (**B**) Violin plots for 10 μm polystyrene beads in deionized water for voltages between the electrodes of 0.0 Vpp (no DEP), 2.0 Vpp, and 2.5 Vpp at a frequency of 1 MHz. The 10 to 90% bounds are plotted as horizontal lines.

One objective of this work is to determine the limits of the optical DEP cytometer device for measuring changes in CMF on a cell-by-cell basis. We employ polystyrene beads in deionized water as a model system. Polystyrene beads with a uniform size were actuated by a DEP sinusoidal voltage at a frequency of 1 MHz. For 10 µm polystyrene beads in deionized water, at 1 MHz, a CMF of ~−0.5 is obtained, with constant hydrodynamic and density properties. Equation (2) gives the force on a particle due to DEP. The force depends linearly on CMF and the square of the applied field and hence also on the square of the applied voltage. This relationship can be used to simulate polystyrene beads with different values of CMF. This effect can be observed in the measured results in Figure 7.

The CMF resolution of our device is estimated by evaluating the distributions of differential velocity for different applied DEP voltages for 10 μm polystyrene beads as shown in Figure 7. For an applied DEP voltage of 0 V (No DEP), the mean of the distribution is near zero, as expected. For 2.0 Vpp, the mean of the distribution is 158 μm/s, and at 2.5 Vpp, the mean is 244 μm/s. In Figure 7B, the data set is shown as violin plots of the differential velocity distributions, with horizontal lines representing the 10th and 90th percentiles. As described previously, a change in applied voltage from 2 Vpp to 2.5 Vpp corresponds to an equivalent change in CMF of 0.18. The 90th percentile of the 2 Vpp is coincident with the 10th percentile of the 2.5 Vpp in this case. Thus, the device’s single-cell CMF discrimination resolution using the 10th and 90th percentiles is 0.18.

### CHO Single-Cell Dielectrophoretic Analysis

**Figure 8:**
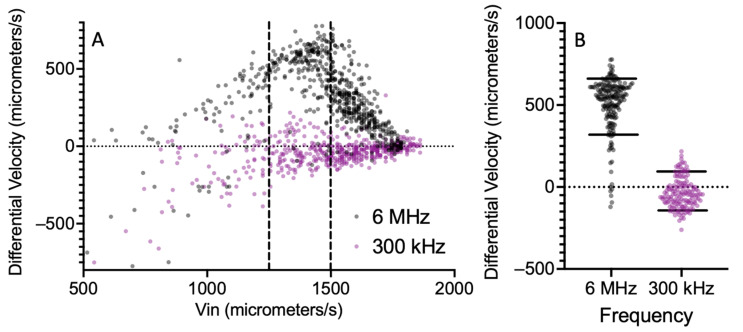
(**A**) Scatter plot of differential velocity versus incoming velocity for CHO cells at an excitation frequency of 6 MHz and 300 kHz in a medium with conductivity 0.17 S/m. The 6 MHz is black and the 300 kHz is purple. (**B**) Distributions over differential velocity for the cells, using cells between the two dashed lines in Figure 8A. The 10th and 90th percentiles are shown as horizontal lines.

A similar approach can be used to estimate the CMF detection resolution for cells. However, in this case, different DEP frequencies are used instead of different DEP voltages. We use CHO cells as an example system, as the CMF of these cells has been extensively studied [25,26,37,38]. For a medium conductivity of 0.17 S/m, viable CHO cells have a CMF frequency spectrum that has both negative (nDEP) and positive (pDEP) ranges, with a cross-over between the two at approximately 450 kHz [37,38]. Figure 8A shows a scatter plot of differential velocity versus incoming velocity for two DEP frequencies (300 kHz and 6 MHz) and a DEP voltage of 8 Vpp. Using the filtering method outlined in Figure 4, cells between the limits of 1250 and 1500 μm/s were chosen. The filtered groups are plotted in Figure 8B. The CMF values corresponding to these two frequencies are −0.04 and +0.31, respectively [38]. This difference is much larger than the discrimination resolution of our device.

**Figure 9:**
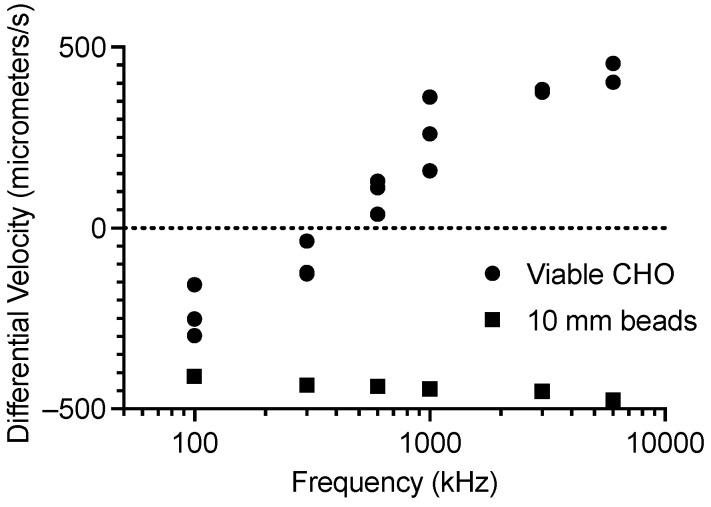
Mean value of differential velocity versus frequency for high-viability CHO cells and polystyrene beads.

For CHO cells, scatter diagrams, such as the one shown in Figure 8A, were gathered at frequencies of 100 kHz, 300 kHz, 600 kHz, 1 MHz, 3 MHz, and 6 MHz. Each scatter diagram point was calculated from 300 to 600 cells. The results were filtered as shown in Figure 8A, and the mean value for each of the filtered groups was calculated. These results are plotted in Figure 9 and are from three separate instances obtained at three different times. There are three points at each frequency, but some overlap. Figure 9 matches the expected CMF for viable CHO cells plotted in Figure 1A. The cross-over frequency is between 300 kHz and 600 kHz, which matches previous measurements using a microwave DEP cytometer [38].

**Figure 10:**
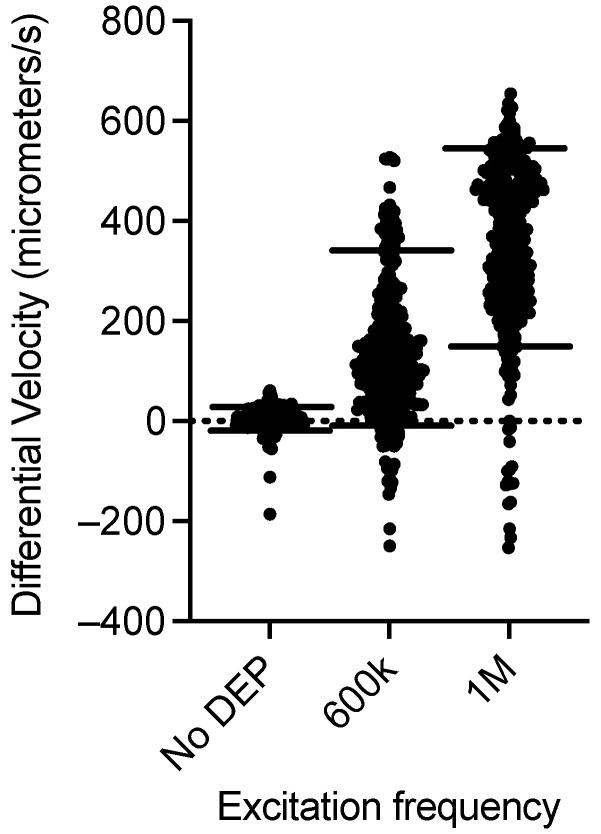
Differential velocity distributions for CHO cells with no excitation and excitation at frequencies of 600 kHz (CMF = 0.11) and 1 MHz (CMF = 0.16). The horizontal line is the 10th to 90th percentile boundary for each distribution.

Similarly to the analysis for polystyrene beads, the CMF discrimination resolution for the CHO model mammalian cell system can be evaluated. The differential velocity distributions for the no DEP, 600 kHz, and 1 MHz excitation cases are plotted in Figure 10. The no DEP case has no excitation voltages applied to the electrodes and hence there is no DEP force on the cells. Therefore, the velocity before and after the electrodes is expected to be the same. Electronic noise, thermal motion, and other effects, such as cell rotation, will lead to uncertainties in the determination of particle velocity [66]. The 10th and 90th percentiles in this case are +22 and −25 μm/s or +1.6 and −1.8% of the 1375 μm/s average velocity. The CMF at 600 kHz is expected to be slightly positive at 0.11 [38]. The mean distribution of the differential velocities for 600 kHz excitation shown in Figure 10 is also slightly positive. However, the gap between the 10th and 90th percentile in the 600 kHz distribution is significantly greater than the no-excitation distribution. Since electronic and other sources of uncertainty are the same for both cases, the variation in the 600 kHz distribution is attributed to the biological cell-to-cell variation in the CMF. From this comparison, we can conclude that the CMF resolution of this approach is sufficient to resolve the inherent CMF variability of CHO cells. At a DEP excitation voltage of 1 MHz, in media of 0.17 S/m, the CMF is expected to be 0.16 [38]. The differential velocity distribution for this condition is also plotted in Figure 10, along with the 10th and 90th percentiles of the distribution. Again, the variation in the distribution can be attributed to the cell-to-cell variation. The distributions for the no-DEP case (with CMF = 0) and the 1 MHz case (CMF ~ 0.16) can be differentiated from each other, even with the cell-to-cell variability. This confirms that our optical cytometer has a CMF discrimination resolution < 0.16.

## 4. Discussion

The aim of using dielectrophoretic-induced differential velocity measurement with an optical array detector was to achieve single-cell dielectric measurement with simultaneous high throughput. Using literature data, simulations were carried out, for example, of applications where dielectric analysis might be used for single-cell discrimination. In the analyses, CMF was used as the measure of relative dielectric change. Some examples of the required CMF discrimination are given in Table 1 and ranged from 0.52 for viability analysis [38] to 0.27 for MDR in cancer [6] to 0.13 for Cl-channel blocking. There are some cases in which dielectric differences are too small to discriminate cell by cell, such as inhibition of ATP production in CHO cells [59]. Differential velocity measurements are less sensitive to the incoming velocity variations due to varying cell height within the channel and also due to variations in cell size. Utilizing the differential approach, CMF discrimination of 0.16 for cells can be accomplished on a cell-by-cell basis. Therefore, the optical cytometer has a CMF discrimination resolution that is suitable for applications such as discrimination of viable from nonviable cells and cancerous from multidrug-resistant cancerous cells.

Our DEP optical cytometer has a reasonable throughput of 5 cells per second. Thus, the full spectral range covering both positive and negative CMF can be gathered in periods of 10 min assuming a sample size of N > 300 for six frequencies (such as that shown in Figure 8). For applications such as viability determination, where DEP analysis at only one frequency is required [21,38], samples of several hundred cells are required, and these can be gathered in a few minutes. Our DEP optical cytometer may also be useful for applications where knowledge of the CMF spectra is needed. For example, DEP is used for cell sorting, but measurement of CMF spectra using a DEP sorting apparatus is very challenging. Our DEP optical cytometer could be used to rapidly find optimal frequencies and medium conductivities.

We note differences when comparing our DEP optical cytometer with other single-cell dielectric analysis methods used to discriminate cell populations. Electrorotation can extract spectra with up to 20 frequencies for a single cell but has a very low throughput of several minutes per cell [30,31,32]. Therefore, extracting spectra for a reasonable population of cells with N > 100 would require several hours. Flow impedance-based devices have high throughput but do not provide single-particle incoming positional or size measures [27]. Lack of size and positional information reduces the cell-to-cell dielectric discrimination resolution. In addition, flow impedance is susceptible to clogging due to the small analysis sensing aperture needed for this method. The dielectric spring method can resolve several frequencies for each cell but has a slightly lower throughput at 1 cell/s [67]. Additionally, the dielectric spring method uses video imaging as a method of acquiring positional information and hence must process large video files. The dielectric spring method could be adapted for use with linear optical array detection, enabling much lower data analysis requirements.

Our DEP optical cytometer has throughput and dielectric discrimination resolution that makes it suitable for a large number of applications, such as cell culture viability monitoring and identification of multidrug-resistant cancer cells. With modest improvements in hardware, the capabilities of the approach can be significantly increased. The throughput can be increased by utilizing a wider channel with a longer optical detection array. An optical array with a 14 μm pitch and length of 28 mm, and corresponding 28 mm wide fluid channels, would increase throughput to close to 100 cells/s. Additionally, 14 μm pitch detectors would allow for the capture of more detailed particle diffraction patterns with commensurate improvements in the estimation of size and potentially other particle optical parameters. Using multiple LED sources and time domain interleaving diffraction patterns would allow two or more wavelengths to be simultaneously acquired. With the addition of a second electrode pair and a second optical array, the dielectric response at a second dielectrophoretic frequency could also be acquired. This later improvement would add additional computational complexity as particles would need to be aligned from one detector array to the next.

## 5. Conclusions

We have successfully developed and demonstrated a novel DEP cytometer based on differential measurement of particle velocity that achieve high throughput. The cytometer employs an array of LED sources in conjunction with a linear optical detector array, enabling high-resolution detection of both attractive and repulsive dielectrophoretic actuation of single cells.

Our experiments with CHO cells show that the acquired dielectrophoretic spectra obtained over the frequency range of 100 kHz to 6 MHz closely align with the CMF spectra obtained using other DEP-based measurement devices.

Utilizing polystyrene beads with diameters of 10 μm and the application of voltage perturbations to simulate equivalent CMF perturbations, we showed our device has a CMF discrimination resolution of less than 0.16 on a single-particle basis. Thus, when the CMF difference between the 10th and 90th percentiles of two populations within a sample of mixed populations exceeds 0.16, our method can accurately assign individual cells to the appropriate population. This could prove useful for applications such as the identification of multidrug-resistant cancer cells. Our device is particularly suited to CHO cell culture viability monitoring, as viable and nonviable cells have a mean difference in CMF of 0.52. In particular, this new technique will be useful for rapidly finding conditions for the optimal dielectric differentiation of cell populations.

Overall, the strength of our method lies in its straightforward apparatus and the utilization of high-throughput, wide, clog-resistant channels. These features contribute to its promising potential for a wide range of applications, where the identification of dielectric populations is important.

## Figures and Tables

**Table 1:** Comparison of the Clausius–Mossotti factor of various cells in different physiological conditions.

Cell Line	Normal	Altered	Medium Conductivity (S/m)	Frequency (MHz)	Condition
Jurkat	0.87	0.47	0.01	15	Apoptotic [17]
Jurkat	0.79	0.24	0.01	15	Anti-cancer drug-treated (DOX-treated) [13]
MCF-7 cell line (human breast)	0.58	0.31	0.0025	15	Multidrug resistance derivatives [6]
Chinese hamster ovary	0.32	−0.2	0.17	6	Apoptotic [19,25]
Chinese hamster ovary	−0.35	−0.37	1.7	6	Stationary phase of fed-batch culture [58]
Chinese hamster ovary	−0.008	−0.1	0.42	6	Inhibition of mitochondria ATP production [59]
Chinese hamster ovary	−0.008	−0.07	0.42	6	Inhibition of ATPase pumps [59]
Human chronic myelogenous leukemia (K562)	0.79	0.9	0.0025	10	Multidrug-resistant leukemic cells (K562AR) [60]
Multidrug-resistant leukemic (K562AR)	0.9	0.77	0.0025	10	Cl^−^ channel blocked with NPPB [60]
Multidrug-resistant leukemic (K562AR)	0.9	0.85	0.0025	10	K^+^ channel blocked with quinine [60]
Human chronic myelogeneous leukemic (K562)	0.79	0.22	0.005	10	Apoptotic [20]

## Data Availability

The data set is available on request from the authors.
